# Effect of Admission Time on the Outcomes of Liver Cirrhosis with Acute Upper Gastrointestinal Bleeding: Regular Hours versus Off-Hours Admission

**DOI:** 10.1155/2018/3541365

**Published:** 2018-11-29

**Authors:** Yingying Li, Bing Han, Hongyu Li, Tingxue Song, Wenchun Bao, Ran Wang, Zhaohui Bai, Kexin Zheng, Qianqian Li, Xiaozhong Guo, Xingshun Qi

**Affiliations:** ^1^Department of Gastroenterology, General Hospital of Shenyang Military Area, Shenyang, 110840, China; ^2^Postgraduate College, Jinzhou Medical University, Jinzhou 121001, China; ^3^Postgraduate College, Liaoning University of Traditional Chinese Medicine, Shenyang, 110840, China; ^4^Postgraduate College, Shenyang Pharmaceutical University, Shenyang 110840, China; ^5^Postgraduate College, Dalian Medical University, Dalian 116044, China

## Abstract

**Background and Aims:**

Acute upper gastrointestinal bleeding (AUGIB) is a lethal complication of liver cirrhosis. We aimed to compare the outcomes of patients with liver cirrhosis and AUGIB who were admitted to hospital on regular hours and off-hours.

**Methods:**

This retrospective study screened all cirrhotic patients with AUGIB who were admitted to our hospital from January 2010 to June 2014 for the test cohort and from December 2014 to March 2018 for the validation cohort. A 1:1 propensity score matching analysis was performed to adjust the Child-Pugh and MELD scores. In-hospital mortality, 5-day rebleeding rate, length of stay, and total payment were primary outcomes.

**Results:**

Overall, 826 and 173 patients with liver cirrhosis and AUGIB were included in the test and validation cohorts, respectively. After propensity score matching, 226 and 40 patients were included in the test and validation cohorts, respectively. The overall analysis of the test cohort found significantly higher Child-Pugh score (P=0.006), 5-day rebleeding rate (18.69% versus 10.72%, P=0.001), and total payment (¥25,906.83 versus ¥22,017.42, P<0.001) in patients admitted on off-hours. By contrast, the overall analysis of the validation cohort did not find any difference in Child-Pugh score, 5-day rebleeding, in-hospital mortality, length of stay, or hospital payment between patients admitted on regular hours and off-hours. Similarly, the propensity score matching analyses of both test and validation cohorts found no difference in these primary outcomes between the two groups.

**Conclusions:**

Off-hours admission might not be negatively associated with the outcomes of patients with liver cirrhosis and AUGIB.

## 1. Introduction

Liver cirrhosis is the 13th major cause of death worldwide. Acute upper gastrointestinal bleeding (AUGIB) is a frequent medical emergency with a high incidence of 45-172/100,000 each year in the general population and is a lethal complication of liver cirrhosis leading to an in-hospital mortality of 10% [1, 2]. Due to the acute performance of AUGIB itself, early diagnosis and timely management are needed. Notably, there are general shortage of staff, a potentially lower professional level of staff, and delayed use of endoscopy during weekends and holidays, which may lead to worse outcomes [3, 4]. Previous studies evaluated the effect of admission time on the outcomes of patients with AUGIB, but their findings were inconsistent. Some authors supported the “weekend effect” that patients admitted during weekends had worse outcomes [5–9], such as higher mortality and rebleeding rate, longer length of stay, and increased cost. On the contrary, others suggested no significant difference in the mortality between patients admitted during weekends and weekdays [10–15]. A meta-analysis [16] reported that off-hours admission was significantly associated with an increased mortality and less timely endoscopy in patients with nonvariceal bleeding but not those with variceal bleeding. More recently, another meta-analysis [17] also had similar results. However, there were some limitations in previous studies.

First, meta-analyses have shown that geographical variation leads to different weekend effect on outcomes [16, 17]. A study, which included 571 patients suspected with upper gastrointestinal bleeding (UGIB) from 8 participating hospitals in the Netherlands [11], reported that patients admitted on weekends had higher mortality and rebleeding rate than those admitted on weekdays. By contrast, two prospective studies [14, 15] conducted in the United Kingdom found no significant difference in mortality of patients with UGIB between weekend and weekday admission groups. A retrospective study conducted in Korea [12], which included 294 cirrhotic patients with acute variceal bleeding, found no significant difference in the in-hospital mortality between weekend and weekday admission groups. Notably, all studies included in the two meta-analyses were not conducted in China mainland. Considering a geographical difference in the staff schedule and management and outcome of AUGIB, further studies should be performed in China mainland.

Second, meta-analyses have also shown that a variation in the source of bleeding leads to different weekend effect on outcomes. All of 4 studies conducted in the United States were based on Nationwide Inpatient Sample, but showed different results [5, 6, 8, 13]. The first study demonstrated that patients with UGIB regardless of source of bleeding admitted on weekends had significantly higher mortality and longer length of stay than those admitted on weekdays [5]. The second study also demonstrated that patients with peptic ulcer hemorrhage admitted on weekends had higher mortality and longer length of stay [6]. The third study further confirmed that patients with nonvariceal UGIB admitted on weekends had higher mortality [8]. However, the fourth study found that the mortality in patients with acute variceal bleeding was similar between weekend and weekday admission groups [13].

Third, previous studies usually compared the effect of weekends versus weekdays on the mortality of AUGIB. But the nighttime during weekdays was often ignored from the definition of off-hours. Thus, further studies should refine the interval of off-hours.

Herein, we performed a retrospective study to compare the outcomes of patients with liver cirrhosis and AUGIB who were admitted to a large tertiary hospital of Northeastern China on regular hours versus off-hours.

## 2. Methods

### 2.1. Study Design

We reviewed the medical records of cirrhotic patients who were consecutively admitted to the General Hospital of Shenyang Military Area from January 2010 to June 2014 as the test cohort. All patients with a diagnosis of liver cirrhosis and AUGIB were eligible. Additionally, we are prospectively collecting all cirrhotic patients who were admitted to our department and underwent contrast-enhanced CT scans and endoscopy since December 2014. Thus, based on the data during the patients' enrollment and follow-up, a validation cohort of cirrhotic patients with AUGIB between December 2014 and March 2018 was established for the present study. Age and sex were not limited. The source of bleeding was not limited. Patients with liver and other malignancies were excluded. Patients with incomplete case information and unavailable electronic medical records were also excluded. Data from repeated admission was not deliberately excluded. The outcomes we observed included 5-day rebleeding rate, in-hospital mortality, length of hospital stay, and total payment during hospitalizations. This study was approved by the Medical Ethical Committee of our hospital and the ethical approval number was k (2017)42. The patient's informed consent was not required in the retrospective study.

### 2.2. Data Collection

The primary data collected were age, sex, admission time, etiology of liver disease, and laboratory tests (i.e., red blood cell, hemoglobin, white blood cell, platelet count, total bilirubin, direct bilirubin, indirect bilirubin, albumin, alanine aminotransferase, aspartate aminotransferase, alkaline phosphatase, gamma-glutamyl transpeptidase, blood urea nitrogen, creatinine, potassium, sodium, prothrombin time, activated partial thromboplastin time, and international normalized ratio [INR]). The severity of esophageal varices was also collected. Treatment options of AUGIB were collected as follows: endoscopic therapy (i.e., band ligation, sclerotherapy, and histoacryl), Sengstaken Blackmore tube, somatostatin and/or octreotide, blood transfusion, proton pump inhibitors (PPIs), and surgery.

### 2.3. Definitions and Formulas

AUGIB was defined as hematemesis and/or melena within 5 days before our admission or positive occult blood test at the day of admission [18]. Regular hours referred to the interval from 8:00 AM to 17:00 PM at the weekdays (i.e., from Monday to Friday). Otherwise, off-hours were considered, and weekends and public holidays were also considered as off-hours. Child-Pugh score [19] was calculated according to hepatic encephalopathy, ascites, total bilirubin, albumin, and INR. Model for end-stage liver disease (MELD) score=9.57 ×  ln⁡  (creatinine  [*μ*mol/L] × 0.01)  + 3.78 ×  ln⁡(bilirubin  [*μ*mol/L] × 0.05) + 11.2 ×  ln ⁡(INR)  + 0.643 [20]. According to the study by Reverter et al. [21], recalibrated MELD score=-5.312+0.207×MELD. Albumin-bilirubin (ALBI) score=-0.085 × albumin (g/L) + 0.66 ×  log_10_⁡bilirubin  (*μ*mol/L) [22, 23].

### 2.4. Statistical Analyses

Continuous variables were reported as median (range) and were compared using the nonparametric Mann-Whitney U test. Categorical variables were reported as frequency (percentage) and were compared using the chi-square test. Subgroup analyses were also conducted based on the presence of varices on endoscopy (AUGIB with endoscopically confirmed varices and without varices on endoscopy). A 1:1 propensity score matching analysis was performed to adjust the effect of gender, age, Child-Pugh score, MELD score, and recalibrated MELD score on the outcomes. A two-tailed P<0.05 was considered statistically significant. All statistical analyses were performed with IBM SPSS 20.0 (IBM Corp.) statistical package and Stata/SE 12.0 (Stata Corp, College Station, TX) software.

## 3. Results

### 3.1. Test Cohort

#### 3.1.1. Patients' Characteristics

Between January 2010 and June 2014, a total of 826 patients with liver cirrhosis and AUGIB were included. Baseline patient characteristics are described in Table 1. Median age was 55.27 years (range: 6.28 to 95.13). Among them, 564 (68.3%) patients were male. Major etiology of liver diseases included hepatitis B virus infection (n=208, 25.2%) and alcohol abuse (n=219, 26.5%). A majority of patients had Child-Pugh class B (339/776, 51.4%). Median MELD score at admission was 6.37 (-7.52 to 38.22). Five hundred and twenty-two patients underwent endoscopy. No, mild, moderate, and severe esophageal varices were observed in 32 (6.1%), 24 (4.6%), 54 (10.3%), and 412 (78.9%) patients, respectively. As for the treatment of AUGIB, 508 (61.5%) patients underwent endoscopic therapy, 20 (2.4%) patients underwent Sengstaken Blackmore tube placement, 750 (90.8%) patients received somatostatin and/or octreotide, 544 (65.9%) patients received blood transfusion, 813 (98.4%) patients received PPIs, and 8 (1.0%) patients underwent surgery. Information regarding 5-day rebleeding was unavailable in 4 patients, because some of their medical records were missing. Five-day rebleeding rate was 14.0% (115/822). In-hospital mortality was 5.7% (47/826). Median length of hospital stay was 11.23 days (range: 0.06 to 100.55). Total payment was ¥23,120.87 (range: 1,287.54 to 226,872.93).

#### 3.1.2. Outcome

Patients admitted on off-hours had lower serum albumin (P<0.001) and higher white blood cell (P<0.001), blood urea nitrogen (P<0.001), potassium (P<0.001), prothrombin time (P=0.034), INR (P=0.040), Child-Pugh score (P=0.006), and ALBI score (P<0.001) than those admitted on regular hours (Table 1). As for the interventions, patients admitted on off-hours had a higher proportion of blood transfusion than those admitted on regular hours (73.3% versus 60.7%, P<0.001). Among the different departments of our hospital, there was no significant difference in the selection of most treatment options for AUGIB between patients admitted on regular hours and off-hours (Supplementary Table 1). As for the outcomes, patients admitted on off-hours had a higher 5-day rebleeding rate (18.7% versus 10.7%, P=0.001) and a larger amount of payment (¥25,906.83 versus ¥22,017.42, P<0.001). In-hospital mortality was not significantly different between the two groups (P=0.418). Length of stay was not significantly different between the two groups (P=0.830).

#### 3.1.3. Subgroup Analyses

The origin of bleeding could be evaluated in 611 patients in the test cohort. They included 591 patients with endoscopically confirmed esophageal and/or gastric varices and 20 patients without varices at endoscopy (Supplementary Table 2).

Among the patients with endoscopically confirmed varices, patients admitted on off-hours were older (P=0.015) and had lower red blood cell (P=0.026) and serum albumin (P<0.001) and higher white blood cell (P<0.001), blood urea nitrogen (P<0.001), potassium (P=0.001), prothrombin time (P=0.027), INR (P=0.04), Child-Pugh score (P<0.001), MELD score (P=0.023), recalibrated MELD score (P=0.023), and ALBI score (P<0.001) than those admitted on regular hours. As for the interventions, patients admitted on off-hours had a higher proportion of blood transfusion (75.5% versus 59.5%, P<0.001) and surgery (2.1% versus 0.3%, P=0.027) than those admitted on regular hours. As for the outcomes, patients admitted on off-hours had a higher 5-day rebleeding rate (16.5% versus 10.6%, P=0.038) and a larger amount of payment (¥29,361.51 versus ¥23,864.24, P<0.001). In-hospital mortality and length of stay were not significantly different between the two groups (P=0.094 and P=0.856, respectively).

Among the patients without varices at endoscopy, no significant difference in demographics, etiology of liver disease, laboratory tests, Child-Pugh score, MELD score, recalibrated MELD score, ALBI score, and treatment options was observed between patients admitted on regular hours and off-hours (P>0.05, in all comparisons). As for the outcomes, none died. Five-day rebleeding rate, length of stay, and total payment were not significantly different between the two groups (P=0.117, P=0.869, and P=0.187, respectively).

#### 3.1.4. Patients' Characteristics after Propensity Score Matching

After a 1:1 propensity score matching analysis, a total of 226 patients with liver cirrhosis and AUGIB were included. Baseline patient characteristics are described in Table 2. Median age was 54.51 years (range: 6.28 to 81.62). Among them, 144 (63.7%) patients were male. Major etiology of liver diseases included hepatitis B virus infection (n=58, 25.7%) and alcohol abuse (n=50, 22. 1%). A majority of patients had Child-Pugh class B (n=121, 53.5%). Median MELD score at admission was 6.12 (-7.14 to 21.56). No, mild, moderate, and severe esophageal varices were observed in 11 (4.9%), 19 (4.4%), 23 (10.2%), and 182 (80.5%) patients, respectively. As for the treatment of AUGIB, 191 (84.5%) patients underwent endoscopic therapy, 8 (3.5%) patients underwent Sengstaken Blackmore tube placement, 218 (96.5%) patients received somatostatin and/or octreotide, 159 (70.4%) patients received blood transfusion, all patients received PPIs, and 1 (0.4%) patient underwent surgery. Five-day rebleeding rate was 14.2% (n=32). In-hospital mortality was 2.2% (n=5). Median length of hospital stay was 12.835 days (range: 2.76 to 78.00). Median total payment was ¥28,633.075 (range: 2,776.55 to 143,048.30).

#### 3.1.5. Outcomes after Propensity Score Matching

After a 1:1 propensity score matching analysis, 113 patients were matched on each group (Table 2). No significant difference in demographics, etiology of liver disease, laboratory tests, Child-Pugh score, MELD score, recalibrated MELD score, ALBI score, and treatment options was observed between the two groups (P>0.05, in all comparisons). As for the outcomes, 5-day rebleeding rate, in-hospital mortality, length of stay, and total payment were not significantly different between the two groups (P=0.445, P=0.651, P=0.229, and P=0.390, respectively).

#### 3.1.6. Subgroup Analyses after Propensity Score Matching

After a 1:1 propensity score matching analysis, 140 patients with endoscopically confirmed varices were matched on each group (Supplementary Table 3). Patients admitted on off-hours had higher white blood cell (P=0.007), blood urea nitrogen (P=0.01), and potassium (P=0.001) than those admitted on regular hours. As for the interventions, patients admitted on off-hours had a higher proportion of blood transfusion (77.1% versus 58.6%, P=0.001) and surgery (2.9% versus 0%, P=0.044) than those admitted on regular hours. As for the outcomes, 5-day rebleeding rate, in-hospital mortality, length of stay, and total payment were not significantly different between the two groups (P=0.306, P=0.409, P=0.421, and P=0.058, respectively).

### 3.2. Validation Cohort

#### 3.2.1. Patients' Characteristics

Between December 2014 and March 2018, a total of 173 patients with liver cirrhosis and AUGIB were included. Baseline patient characteristics are described in Table 3. Median age was 56.60 years (range: 20.57 to 88.73). Among them, 121 (69.9%) patients were male. Major etiology of liver diseases included hepatitis B virus infection (n=47, 27.2%) and alcohol abuse (n=55, 31.8%). A majority of patients had Child-Pugh class B (94/169, 55.6%). Median MELD score at admission was 7.22 (-3.16 to 23.19). One hundred and fifty-one patients underwent endoscopy. No, mild, moderate, and severe esophageal varices were observed in 9 (6.0%), 23 (15.2%), 26 (17.2%), and 93 (61.6%) patients, respectively. The origin of bleeding could be evaluated in 162 patients, of whom only 4 did not have esophageal and/or gastric varices. As for the treatment of AUGIB, 139 (80.3%) patients underwent endoscopic therapy, 1 (0.6%) patients underwent Sengstaken Blackmore tube placement, 160 (92.5%) patients received somatostatin and/or octreotide, 88 (50.9%) patients received blood transfusion, 173 (100%) patients received PPIs, and 0 (0%) patients underwent surgery. Information regarding 5-day rebleeding was unavailable in one patient. Five-day rebleeding rate was 7.6% (13/172). In-hospital mortality was 2.3% (4/173). Median length of hospital stay was 10.10 days (range: 0.12 to 32.94). Total payment was ¥24,328.31 (range: 3,427.24 to 98,215.78).

#### 3.2.2. Outcomes

No significant difference in demographics, etiology of liver disease, laboratory tests, Child-Pugh score, MELD score, recalibrated MELD score, ALBI score, and treatment options was observed between the two groups (P>0.05, in all comparisons). As for the outcomes, 5-day rebleeding rate, in-hospital mortality, length of stay, and total payment were not significantly different between the two groups (P=0.579, P=0.973, P=0.335, and P=0.166, respectively) (Table 3).

#### 3.2.3. Patients' Characteristics after Propensity Score Matching

After a 1:1 propensity score matching analysis, a total of 40 patients with liver cirrhosis and AUGIB were included. Baseline patient characteristics are described in Table 4. Median age was 56.86 years (range: 20.57 to 75.64). Among them, 28 (70%) patients were male. Major etiology of liver diseases included hepatitis B virus infection (n=9, 22.5%) and alcohol abuse (n=14, 35%). A majority of patients had Child-Pugh class B (n=22, 55%). Median MELD score at admission was 5.82 (range: -2.38 to 23.19). No, mild, moderate, and severe esophageal varices were observed in 2 (5%), 5 (12.5%), 6 (15%), and 27 (67.5%) patients, respectively. As for the treatment of AUGIB, 38 (95%) patients underwent endoscopic therapy, no patient underwent Sengstaken Blackmore tube placement, 39 (97.5%) patients received somatostatin and/or octreotide, 21 (52.5%) patients received blood transfusion, all patients received PPIs, and no patient underwent surgery. Five-day rebleeding rate was 12.5% (n=5). In-hospital mortality was 2.5% (n=1). Median length of hospital stay was 11.95 days (range: 5.73 to 31.06). Median total payment was ¥24,961.33 (range: 11,212.15 to 81,125.52).

#### 3.2.4. Outcomes after Propensity Score Matching

After a 1:1 propensity score matching analysis, 20 patients were matched on each group (Table 4). No significant difference in demographics, etiology of liver disease, laboratory tests, Child-Pugh score, MELD score, recalibrated MELD score, ALBI score, and treatment options was observed between the two groups (P>0.05, in all comparisons). As for the outcomes, 5-day rebleeding rate, in-hospital mortality, length of stay, and total payment were not significantly different between the two groups (P=0.633, P=0.311, P=0.441, and P=0.829, respectively).

## 4. Discussion

Traditionally, a worse outcome in patients with UGIB during the weekend was potentially attributed to lower staffing levels and relatively younger and inexperienced staff [11]. Indeed, both overall analyses and subgroup analyses of patients with endoscopically confirmed varices in the test cohort demonstrated a significantly higher 5-day rebleeding rate and a larger amount of payment in patients admitted on off-hours. This might be primarily due to worse liver dysfunction in patients admitted on off-hours, such as lower albumin and higher prothrombin time, INR, Child-Pugh score, and ALBI score. As the Child-Pugh score was matched, the propensity score matching analyses of both test and validation cohorts showed no significant effect of admission time on the rebleeding rate, in-hospital mortality, length of stay, and total payment of cirrhotic patients with AUGIB. These findings suggested that the weekend effect might not be an independent risk factor for worse outcomes of cirrhotic patients with AUGIB.

A meta-analysis [16] found that off-hours admission was not associated with a higher risk of rebleeding rate (OR=1.06, 95%CI=0.83-1.35, and P=0.66) and longer length of stay (WMD 0.06 day, 95%CI=-0.30 - -0.42, P=0.747). These previous findings were consistent with our results regarding 5-day rebleeding rate and length of stay. Notably, our study focused on the 5-day rebleeding rate after treatment during hospitalization. However, the interval of rebleeding was not specified in the meta-analysis. As we have known, the length of stay was usually associated with the severity of illness [24]. In addition, as well known, liver dysfunction as estimated by Child-Pugh score and MELD score were risk factors for mortality of cirrhotic patients with AUGIB [25–27].

Two of the published meta-analyses [16, 17] demonstrated a significant weekend effect on the mortality in patients with nonvariceal UGIB, but not those with variceal bleeding. This finding seemed to be consistent with our results regarding mortality. Notably, our study focused on the outcomes during hospitalization, but not those after discharge. The fact is readily understood that admission time mainly influenced the in-hospitalization outcomes but marginally influenced the outcomes after discharge. Indeed, a meta-analysis also suggested that off-hours admission negatively influenced in-hospital mortality (P=0.009), rather than 30-day mortality (P=0.116).

Our study has the following advantages. First, we refined the definitions of off-hours admission. Second, we included a test cohort and a validation cohort which can reduce the sampling bias to some extent. Third, we employed a propensity score matching analysis. Thus, the patient characteristics, especially Child-Pugh and MELD scores which are significantly associated with prognosis of liver cirrhosis, are comparable between the two groups. Our results become more stable. However, the major drawback of our study should be that not all patients undergo endoscopy to evaluate the source of bleeding. Additionally, the sample size is not adequate in the validation cohort. Finally, a potential selection bias could not be neglected due to the retrospective nature of this study.

In conclusion, off-hours admission might not be associated with outcomes in patients with liver cirrhosis and AUGIB. However, the geographical difference should not be neglected to extrapolate our findings.

## Figures and Tables

**Table 1:** Characteristics of patients with liver cirrhosis and AUGIB in test cohort.

**Variables**	**No. Pts**	**Overall**	**No. Pts**	**Regular hours**	**No. Pts**	**Off-hours**	**P value**
Age (years)	826	55.27 (6.28-95.13)	486	54.54 (20.88-95.13)	340	55.95 (6.28-84.56)	0.074
Sex (male)	826	564 (68.3%)	486	337 (69.3%)	340	227 (66.8%)	0.434
Etiology of Liver Diseases	826		486		340		0.387
HBV		208 (25.2%)		126 (25.9%)		82 (24.1%)	
HCV		51 (6.2%)		32 (6.6%)		19 (5.6%)	
HBV + HCV		6 (0.7%)		2 (0.4%)		4 (1.2%)	
Alcohol Abuse		219 (26.5%)		125 (25.7%)		94 (27.7%)	
HBV + Alcohol Abuse		64 (7.7%)		42 (8.6%)		22 (6.5%)	
HCV + Alcohol Abuse		13 (1.6%)		7 (1.4%)		6 (1.8%)	
HBV + HCV + Alcohol Abuse		2 (0.2%)		1 (0.2%)		1 (0.3%)	
Drug Related		35 (4.2%)		23 (4.7%)		12 (3.5%)	
Autoimmune Liver Diseases		56 (6.8%)		26 (5.4%)		30 (8.8%)	
Other or Unclear Etiology		172 (20.8%)		102 (21%)		70 (20.6%)	
Laboratory Tests							
Red Blood Cell (10^12^/L)	822	2.56 (0.93-5.49)	482	2.61 (0.93-5.49)	340	2.49 (1.05-5.10)	0.250
Hemoglobin (g/L)	822	72.00 (23.00-180.00)	482	73.00 (23.00-164.00)	340	71.00 (23.00-180.00)	0.512
White Blood Cell (10^9^/L)	822	4.70 (0.40-46.10)	482	4.20 (0.80-33.50)	340	5.30 (0.90-46.10)	<0.001
Platelet Count (10^9^/L)	822	75.00 (9.00-842.00)	482	74.00 (9.00-842.00)	340	77.50 (17.00-775.00)	0.429
Total Bilirubin (umol/L)	820	20.30 (3.30-679.10)	480	20.10 (4.10-679.10)	340	20.50 (3.30-250.80)	0.598
Direct Bilirubin (umol/L)	820	8.30 (0.50-413.80)	480	8.20 (1.30-413.80)	340	8.50 (0.50-195.20)	0.916
Indirect Bilirubin (umol/L)	820	11.75 (1.30-265.30)	480	11.50 (1.30-265.30)	340	12.05 (2.00-126.60)	0.436
Albumin (g/L)	796	30.40 (9.60-49.30)	469	31.00 (9.60-49.30)	327	29.30 (10.00-48.00)	<0.001
Alanine Aminotransferase (U/L)	818	23.00 (5.00-1064.00)	479	23.00 (5.00-730.00)	339	24.00 (5.00-1064.00)	0.403
Aspartate Aminotransferase (U/L)	818	31.00 (7.00-1487.00)	479	31.00 (7.00-1399.00)	339	32.00 (8.00-1487.00)	0.255
Alkaline Phosphatase (U/L)	818	73.00 (1.30-889.00)	479	74.00 (17.44-889.00)	339	72.00 (1.30-688.00)	0.535
Gamma-glutamyl Transpeptidase (U/L)	818	35.00 (5.00-1168.00)	479	35.00 (5.00-1168.00)	339	37.00 (6.00-755.00)	0.945
Blood Urea Nitrogen (mmol/L)	792	7.71 (1.58-42.83)	465	7.06 (1.58-42.83)	327	8.21 (1.96-37.67)	<0.001
Serum Creatinine (umol/L)	791	61.00 (20.00-919.00)	465	60.00 (20.00-919.00)	326	61.00 (24.00-327.00)	0.284
Potassium (mmol/L)	810	4.06 (2.13-7.87)	473	4.00 (2.13-7.07)	337	4.12 (2.79-7.78)	<0.001
Sodium (mmol/L)	810	138.80 (83.00-160.80)	473	139.10 (83.00-160.80)	337	138.50 (116.40-152.40)	0.055
PT (seconds)	794	16.20 (10.80-62.80)	465	15.90 (10.80-62.80)	329	16.50 (11.00-49.50)	0.034
APTT (seconds)	791	40.30 (25.70-180.00)	463	40.50 (25.7-180.00)	328	39.95 (27.30-97.20)	0.452
INR	791	1.31 (0.77-7.96)	463	1.28 (0.77-7.96)	328	1.35 (0.79-5.94)	0.040
Child-Pugh Score	776	7.00 (5.00-15.00)	455	7.00 (5.00-15.00)	321	7.00 (5.00-15.00)	0.006
Child-Pugh A/B/C	776	234 (30.2%)/339 (51.4%)/143 (18.4%)	455	160 (35.2%)/216 (47.5%)/79 (17.3%)	321	74 (23.1%)/183 (57.0%)/64 (19.9%)	0.001
MELD Score	772	6.37 (-7.52-38.22)	453	5.87 (-7.52-38.22)	319	7.06 (-7.44-37.65)	0.067
Recalibrated MELD Score	772	-4.00 (-6.87-2.60)	453	-4.10 (-6.87-2.60)	319	-3.85 (-6.85-2.48)	0.067
ALBI Score	793	-1.70 (-3.31-0.22)	467	-1.77 (-3.31-0.03)	326	-1.59 (-3.13-0.22)	<0.001
Esophageal Varices (No/Mild/Moderate/Severe)	522	32 (6.1%)/24 (4.6%)/ 54 (10.3%)/412 (78.9%)	309	20 (6.5%)/17 (5.5%)/ 32(10.4%)/240 (77.6%)	213	12 (5.6%)/7 (3.3%)/ 22 (10.3%)/172 (80.8%)	0.650
Treatment							
Endoscopic Treatment	826	508 (61.5%)	486	310 (63.8%)	340	198 (58.2%)	0.107
Sengstaken Blakemore	826	20 (2.4%)	486	12 (2.5%)	340	8 (2.4%)	0.915
Somatostatin and/or Octreotide	826	750 (90.8%)	486	435 (80.5%)	340	315 (92.7%)	0.124
Blood Transfusion	826	544 (65.9%)	486	295 (60.7%)	340	249 (73.3%)	<0.001
PPIs	826	813 (98.4%)	486	477 (98.2%)	340	336 (98.8%)	0.443
Surgery	826	8 (1.0%)	486	2 (0.4%)	340	6 (1.8%)	0.051
5-day Re-bleeding After Treatment	822	115 (14.0%)	485	52 (10.7%)	337	63 (18.7%)	0.001
Death During Hospitalization	826	47 (5.7%)	486	25 (5.1%)	340	22(6.5%)	0.418
Length of Stay (days)	826	11.23 (0.06-100.55)	486	11.27 (0.06-78.00)	340	11.13 (0.09-100.55)	0.830
Total Payment (¥)	826	23,120.87 (1,287.54-226,872.93)	486	22,017.42 (1,683.37-126,413.58)	340	25,906.83 (1,287.54-226,872.93)	<0.001

Data are expressed as median (range) or frequency (percentage).

*Abbreviations.* AUGIB: acute upper gastrointestinal bleeding; HBV: Hepatitis B virus; HCV: Hepatitis C virus; PT: prothrombin time; APTT: activated partial thromboplastin time; INR: international normalized ratio; MELD: model for end-stage liver disease; ALBI: albumin-bilirubin; PPIs: proton pump inhibitors; ¥: Renminbi.

**Table 2:** Characteristics of patients with liver cirrhosis and AUGIB after propensity matching in test cohort.

**Variables**		**Propensity matching patients**		**P value**
	**Overall (n=226)**	**Regular hours (n=113)**	**Off-hours (n=113)**	
Age (years)	54.51 (6.28-81.62)	54.41 (29.85-81.62)	54.60 (6.28-78.66)	0.829
Sex (male)	144 (63.7%)	73 (64.6%)	71 (62.8%)	0.782
Etiology of Liver Diseases				0.612
HBV	58 (25.7%)	29 (25.7%)	29 (25.7%)	
HCV	14 (6.2%)	7 (6.2%)	7 (6.2%)	
HBV+ HCV	1 (0.4%)	1 (0.9%)	0 (0%)	
Alcohol Abuse	50 (22.1%)	26 (23.0%)	24 (21.2%)	
HBV+ Alcohol Abuse	26 (11.5%)	15 (13.3%)	11 (9.7%)	
HCV+ Alcohol Abuse	3 (1.3%)	0 (0%)	3 (2.7%)	
HBV+ HCV+ Alcohol Abuse	1 (0.4%)	0 (0%)	1 (0.9%)	
Drug Related	10 (4.4%)	5 (4.4%)	5 (4.4%)	
Autoimmune Liver Diseases	12 (5.3%)	5 (4.4%)	7 (6.2%)	
Other or Unclear Etiology	51 (22.6%)	25 (22.1%)	26 (23.0%)	
Laboratory Tests				
Red Blood Cell (10^12^/L)	2.54 (0.98-5.49)	2.60 (0.98-5.49)	2.53 (1.15-4.68)	0.989
Hemoglobin (g/L)	71.50 (23.00-157.00)	72.00 (23.00-157.00)	71.00 (23.00-133.00)	0.901
White Blood Cell (10^9^/L)	4.30 (1.00-26.30)	4.10 (1.10-26.30)	4.40 (1.00-17.50)	0.239
Platelet Count (10^9^/L)	74.00 (17.00-435.00)	72.00 (27.00-303.00)	79.00 (17.00-435.00)	0.538
Total Bilirubin (umol/L)	18.80 (3.30-187.40)	19.00 (6.10-90.40)	18.40 (3.30-187.40)	0.669
Direct Bilirubin (umol/L)	7.50 (0.50-151.60)	7.50 (1.30-56.30)	7.20 (0.50-151.60)	0.384
Indirect Bilirubin (umol/L)	11.35 (2.40-56.80)	11.20 (3.20-41.10)	11.40 (2.40-56.80)	0.771
Albumin (g/L)	30.10 (10.00-49.30)	30.48 (10.50-49.30)	29.40 (10.00-45.60)	0.580
Alanine Aminotransferase (U/L)	23.00 (5.00-234.00)	23.00 (6.00-234.00)	23.00 (5.00-154.00)	0.896
Aspartate Aminotransferase (U/L)	31.00 (9.00-263.00)	31.00 (12.00-263.00)	31.00 (9.00-175.00)	0.872
Alkaline Phosphatase (U/L)	74.00 (17.44-707.00)	77.00 (17.44-707.00)	71.00 (21.00-685.00)	0.282
Gamma-glutamyl Transpeptidase (U/L)	36.00 (8.00-1168.00)	45.00 (8.00-1168.00)	33.00 (9.00-737.00)	0.354
Blood Urea Nitrogen (mmol/L)	7.62 (1.96-34.00)	7.66 (2.04-34.00)	7.61 (1.96-28.10)	0.989
Serum Creatinine (umol/L)	60.00 (28.00-234.00)	60.00 (28.00-225.70)	59.00 (30.50-234.00)	0.655
Potassium (mmol/L)	4.06 (2.90-5.50)	4.05 (3.00-5.50)	4.08 (2.90-5.50)	0.285
Sodium (mmol/L)	138.50 (128.00-150.00)	138.30 (130.40-148.00)	138.50 (128.00-150.00)	0.773
PT (seconds)	15.80 (11.00-46.30)	15.60 (12.10-36.10)	16.00 (11.00-46.30)	0.912
APTT (seconds)	39.55 (27.30-66.80)	40.10 (28.20-66.80)	38.50 (27.30-63.90)	0.253
INR	1.28 (0.79-4.77)	1.26 (0.90-3.57)	1.29 (0.79-4.77)	0.950
Child-Pugh Score	7.00 (5.00-13.00)	7.00 (5.00-12.00)	7.00 (5.00-13.00)	0.891
Child-Pugh A/B/C	72 (31.9%)/121 (53.5%)/33 (14.6%)	35 (31.0%)/64 (56.6%)/14 (12.4%)	37 (32.7%)/57 (50.5%)/19 (16.8%)	0.544
MELD Score	6.12 (-7.14-21.56)	6.12 (-5.03-20.16)	5.68 (-7.14-21.56)	0.733
Recalibrated MELD Score	-4.05 (-6.79 - -0.85)	-4.04 (-6.35 - -1.14)	-4.14 (-6.79 - -0.85)	0.733
ALBI Score	-1.70 (-3.23-0.22)	-1.70 (-3.23 - -0.43)	-1.69 (-3.06-0.22)	0.767
Esophageal Varices (No/Mild/Moderate/Severe)	11 (4.9%)/10 (4.4%)/23 (10.2%)/182 (80.5%)	5 (4.4%)/5 (4.4%)/11 (9.8%)/92 (81.4%)	6 (5.3%)/5 (4.4%)/12 (10.6%)/90 (79.7%)	0.984
Treatment				
Endoscopic Treatment	191 (84.5%)	96 (85.0%)	95 (84.1%)	0.854
Sengstaken Blakemore	8 (3.5%)	5 (4.4%)	3 (2.7%)	0.472
Somatostatin and/or Octreotide	218 (96.5%)	107 (94.7%)	111 (98.2%)	0.150
Blood Transfusion	159 (70.4%)	80 (70.8%)	79 (69.9%)	0.884
PPIs	226 (100%)	113 (100%)	113 (100%)	NA
Surgery	1 (0.4%)	1 (0.9%)	0 (0%)	0.316
5-day Re-bleeding After Treatment	32 (14.2%)	14 (12.4%)	18 (15.9%)	0.445
Death During Hospitalization	5 (2.2%)	2 (1.8%)	3 (2.7%)	0.651
Length of Stay (days)	12.835 (2.76-78.00)	12.97 (3.91-78.00)	12.41 (2.76-45.26)	0.229
Total Payment (¥)	28,633.075 (2,776.55-143,048.30)	26,733.81 (2,776.55-115,201.67)	29,361.51 (5,856.09-143,048.30)	0.390

Data are expressed as median (range) or frequency (percentage).

*Abbreviations*. AUGIB: acute upper gastrointestinal bleeding; HBV: Hepatitis B virus; HCV: Hepatitis C virus; PT, prothrombin time; APTT: activated partial thromboplastin time; INR: international normalized ratio; MELD: model for end-stage liver disease; ALBI: albumin-bilirubin; PPIs: Proton pump inhibitors; ¥, Renminbi.

**Table 3:** Characteristics of patients with liver cirrhosis and AUGIB in validation cohort.

**Variables**	**No. Pts**	**Overall**	**No. Pts**	**Regular hours**	**No. Pts**	**Off-hours**	**P value**
Age (years)	173	56.60 (20.57-88.73)	131	56.60 (20.57-75.73)	42	56.56 (33.42-88.73)	0.683
Sex (male)	173	121 (69.9%)	131	91 (69.5%)	42	30 (71.4%)	0.809
Etiology of Liver Diseases	173		131		42		0.830
HBV		47 (27.2%)		37 (28.2%)		10 (23.8%)	
HCV		17 (9.8%)		12 (9.2%)		5 (11.9%)	
Alcohol Abuse		55 (31.8%)		41 (31.3%)		14 (33.3%)	
HBV + Alcohol Abuse		3 (1.7%)		2 (1.5%)		1 (2.4%)	
HBV + HCV + Alcohol Abuse		1 (0.6%)		1 (0.8%)		0 (0%)	
Drug Related		1 (0.6%)		1 (0.8%)		0 (0%)	
Autoimmune Liver Diseases		3 (1.7%)		3 (2.3%)		0 (0%)	
Other or Unclear Etiology		46 (26.6%)		34 (25.9%)		12 (28.6%)	
Laboratory Tests							
Red Blood Cell (10^12^/L)	173	2.70 (1.32-5.11)	131	2.70 (1.32-5.11)	42	2.62 (1.56-4.14)	0.372
Hemoglobin (g/L)	173	73.00 (31.00-156.00)	131	73.00 (31.00-156.00)	42	73.50 (38.00-130.00)	0.575
White Blood Cell (10^9^/L)	173	4.20 (1.00-23.10)	131	4.10 (1.00-23.10)	42	4.35 (1.70-14.90)	0.368
Platelet Count (10^9^/L)	173	71.00 (18.00-457.00)	131	73.00 (18.00-377.00)	42	68.50 (23.00-457.00)	0.980
Total Bilirubin (umol/L)	172	20.80 (5.20-210.00)	131	21.60 (5.20-210.00)	41	18.30 (6.70-106.10)	0.587
Direct Bilirubin (umol/L)	172	8.70 (2.00-146.90)	131	9.00 (2.00-146.90)	41	8.30 (2.50-60.10)	0.643
Indirect Bilirubin (umol/L)	172	10.65 (2.60-79.90)	131	11.40 (2.60-69.20)	41	9.80 (4.20-79.90)	0.801
Albumin (g/L)	171	29.40 (17.00-50.70)	130	29.65 (17.20-50.70)	41	29.40 (17.00-41.00)	0.693
Alanine Aminotransferase (U/L)	172	20.70 (5.00-275.36)	131	20.98 (6.00-140.00)	41	17.17 (5.00-275.36)	0.504
Aspartate Aminotransferase (U/L)	172	27.00 (9.63-310.39)	131	27.50 (9.63-278.00)	41	26.11 (10.00-310.39)	0.801
Alkaline Phosphatase (U/L)	172	78.23 (24.02-378.66)	131	75.66 (24.02-378.66)	41	79.75 (46.24-197.00)	0.981
Gamma-glutamyl Transpeptidase (U/L)	172	31.98 (7.54-1227.00)	131	29.61 (7.54-1227.00)	41	40.53 (7.69-295.00)	0.346
Blood Urea Nitrogen (mmol/L)	172	7.60 (2.17-47.25)	131	7.40 (2.17-47.25)	41	7.96 (2.47-20.15)	0.993
Serum Creatinine (umol/L)	172	69.145 (34.40-267.63)	131	67.03 (34.40-267.63)	41	74.21 (34.90-144.98)	0.116
Potassium (mmol/L)	173	3.95 (2.48-5.54)	131	3.95 (2.48-5.54)	42	3.97 (3.12-5.49)	0.876
Sodium (mmol/L)	173	138.50 (124.00-152.90)	131	138.40 (130.40-152.90)	42	138.80 (124.00-145.70)	0.544
PT (seconds)	171	16.10 (10.50-27.40)	131	16.10 (11.60-27.40)	40	16.05 (10.50-27.20)	0.606
APTT (seconds)	170	37.85 (19.40-59.70)	131	37.90 (27.50-59.70)	39	37.40 (19.40-52.10)	0.542
INR	170	1.32 (0.91-2.55)	131	1.33 (1.01-2.55)	39	1.31 (0.91-2.50)	0.681
Child-Pugh Score	169	8.00 (5.00-15.00)	130	8.00 (5.00-12.00)	39	7.00 (5.00-12.00)	0.603
Child-Pugh A/B/C	169	51 (30.2%)/94 (55.6%) /24 (14.2%)	130	37 (28.5%)/75 (57.7%) /18 (13.8%)	39	14 (35.9%)/19 (48.7%) /6 (15.4%)	0.596
MELD Score	170	7.22 (-3.16-23.19)	131	7.26 (-3.16-20.70)	39	7.22 (-2.38-23.19)	0.671
Recalibrated MELD Score	170	-3.82 (-5.97- -0.51)	131	-3.81 (-5.97- -1.03)	39	-3.82 (-5.80- -0.51)	0.671
ALBI Score	171	-1.63 (-3.40- -0.11)	130	-1.62 (-3.40- -0.11)	41	-1.68 (-2.70- -0.49)	0.675
Esophageal Varices (No/Mild/Moderate/Severe)	151	9 (6.0%)/23 (15.2%)/26 (17.2%)/93 (61.6%)	116	6 (5.2%)/18 (15.5%)/20 (17.2)/72 (61.1%)	35	3 (8.6%)/5 (14.3%)/6 (17.1%)/21 (60.0%)	0.965
Treatment							
Endoscopic Treatment	173	139 (80.3%)	131	104 (79.4%)	42	35 (83.3%)	0.576
Sengstaken Blakemore	173	1 (0.6%)	131	0 (0%)	42	1 (2.4%)	0.077
Somatostatin and/or Octreotide	173	160 (92.5%)	131	119 (90.8%)	42	41 (97.6%)	0.147
Blood Transfusion	173	88 (50.9%)	131	62 (47.3%)	42	26 (61.9%)	0.100
PPIs	173	173 (100%)	131	131 (100%)	42	42 (100%)	NA
Surgery	173	0 (0%)	131	0 (0%)	42	0 (0%)	NA
5-day Re-bleeding After Treatment	172	13 (7.6%)	130	9 (6.9%)	42	4 (9.5%)	0.579
Death During Hospitalization	173	4 (2.3%)	131	3 (2.3%)	42	1 (2.4%)	0.973
Length of Stay (days)	173	10.10 (0.12-32.94)	131	10.10 (0.12-32.94)	42	10.28 (5.02-31.06)	0.335
Total Payment (¥)	173	24,328.31 (3,427.24-98,215.78)	131	22,685.20 (3,427.24-98,215.78)	42	25,213.81 (9,970.37-81,125.52)	0.166

Data are expressed as median (range) or frequency (percentage).

*Abbreviations*. AUGIB: acute upper gastrointestinal bleeding; HBV: Hepatitis B virus; HCV: Hepatitis C virus; PT: prothrombin time; APTT: activated partial thromboplastin time; INR: international normalized ratio; MELD: model for end-stage liver disease; ALBI: albumin-bilirubin; PPIs: proton pump inhibitors; ¥: Renminbi.

**Table 4:** Characteristics of patients with liver cirrhosis and AUGIB after propensity matching in validation cohort.

**Variables**		**Propensity matching patients**		**P value**
	**Overall (n=40)**	**Regular hours (n=20)**	**Off-hours (n=20)**	
Age (years)	56.86 (20.57-75.64)	60.64 (20.57-75.64)	55.52 (35.18-74.33)	0.160
Sex (male)	28 (70%)	13 (65%)	15 (75%)	0.490
Etiology of Liver Diseases				0.622
HBV	9 (22.5%)	4 (20%)	5 (25%)	
HCV	4 (10%)	2 (10%)	2 (10%)	
Alcohol Abuse	14 (35%)	7 (35%)	7 (35%)	
Drug Related	2 (5%)	0 (0%)	2 (10%)	
Autoimmune Liver Diseases	1 (2.5%)	1 (5%)	0 (0%)	
Other or Unclear Etiology	10 (25%)	6 (30%)	4 (20%)	
Laboratory Tests				
Red Blood Cell (10^12^/L)	2.625 (1.65-3.89)	2.58 (1.69-3.89)	2.705 (1.65-3.78)	0.968
Hemoglobin (g/L)	74.50 (38.00-126.00)	77.00 (53.00-122.00)	72.50 (38.00-126.00)	0.473
White Blood Cell (10^9^/L)	4.40 (1.80-14.90)	4.40 (2.20-11.70)	4.35 (1.80-14.90)	0.839
Platelet Count (10^9^/L)	72.50 (27.00-301.00)	72.50 (27.00-301.00)	72.00 (32.00-145.00)	0.978
Total Bilirubin (umol/L)	18.10 (5.50-106.10)	16.85 (5.50-50.90)	18.10 (8.50-106.10)	0.256
Direct Bilirubin (umol/L)	7.85 (2.20-60.10)	7.10 (2.20-41.50)	7.95 (3.00-60.10)	0.675
Indirect Bilirubin (umol/L)	9.50 (2.80-46.00)	8.55 (2.80-22.10)	10.00 (5.40-46.00)	0.148
Albumin (g/L)	30.65 (18.00-39.20)	30.85 (19.00-38.60)	30.00 (18.00-39.20)	1
Alanine Aminotransferase (U/L)	21.805 (6.79-88.77)	21.805 (9.44-72.47)	20.415 (6.79-88.77)	0.756
Aspartate Aminotransferase (U/L)	25.635 (10.74-136.00)	28.38 (11.00-88.79)	25.635 (10.74-136.00)	0.978
Alkaline Phosphatase (U/L)	80.10 (40.34-234.00)	80.94 (40.34-234.00)	78.245 (46.24-149.45)	0.705
Gamma-glutamyl Transpeptidase (U/L)	41.245 (7.69-295.00)	40.48 (13.71-254.00)	42.765 (7.69-295.00)	0.935
Blood Urea Nitrogen (mmol/L)	7.395 (2.98-18.83)	7.055 (2.98-18.83)	8.40 (4.65-18.72)	0.245
Serum Creatinine (umol/L)	70.11 (34.90-143.10)	66.68 (42.60-95.20)	74.255 (34.90-143.10)	0.117
Potassium (mmol/L)	3.95 (3.10-4.66)	3.88 (3.10-4.66)	3.98 (3.27-4.59)	0.330
Sodium (mmol/L)	139.10 (124.00-145.70)	138.50 (135.00-141.40)	139.45 (124.00-145.70)	0.279
PT (seconds)	15.80 (13.30-24.50)	15.40 (13.30-23.90)	16.05 (13.50-24.50)	0.417
APTT (seconds)	36.25 (30.80-47.50)	35.90 (30.80-45.20)	37.15 (32.20-47.50)	0.351
INR	1.275 (1.02-2.19)	1.255 (1.08-2.08)	1.31 (1.02-2.19)	0.516
Child-Pugh Score	8.00 (5.00-12.00)	8.00 (5.00-12.00)	7.00 (5.00-12.00)	0.836
Child-Pugh A/B/C	14 (35%)/22 (55%)/4 (10%)	7 (35%)/12 (60%)/1 (5%)	7 (35%)/10 (50%)/3 (15%)	0.544
MELD Score	5.82 (-2.38-23.19)	5.51 (-1.04-14.75)	6.51 (-2.38-23.19)	0.245
Recalibrated MELD Score	-4.11 (-5.80- -0.51)	-4.17 (-5.53- -2.26)	-3.96 (-5.80- -0.51)	0.245
ALBI Score	-1.67 (-2.62- -0.49)	-1.73 (-2.44- -0.49)	-1.67 (-2.62- -0.60)	0.787
Esophageal Varices (No/Mild/Moderate/Severe)	2 (5%)/5 (12.5%)/6 (15%)/27 (67.5%)	1 (5%)/3 (15%)/3 (15%)/13 (65%)	1 (5%)/2 (10%)/3 (15%)/14 (70%)	0.971
Treatment				
Endoscopic Treatment	38 (95%)	19 (95%)	19 (95%)	1
Sengstaken Blakemore	0 (0%)	0(0%)	0(0%)	NA
Somatostatin	39 (97.5%)	20 (100%)	19 (95%)	0.311
Blood Transfusion	21 (52.5%)	9 (45%)	12 (60%)	0.342
PPIs	40 (100%)	20 (100%)	20 (100%)	NA
Surgery	0 (0%)	0 (0%)	0 (0%)	NA
5-day Re-bleeding After Treatment	5 (12.5%)	2 (10%)	3 (15%)	0.633
Death During Hospitalization	1 (2.5%)	0 (0%)	1 (5%)	0.311
Length of Stay (days)	11.95 (5.73-31.06)	11.95 (5.73-21.95)	11.28 (7.52-31.06)	0.441
Total Payment (¥)	24,961.33 (11,212.15-81,125.52)	25,985.45 (15,513.68-56,952.12)	24,961.33 (11,212.15-81,125.52)	0.829

Data are expressed as median (range) or frequency (percentage).

*Abbreviations*. AUGIB: acute upper gastrointestinal bleeding; HBV: Hepatitis B virus; HCV: Hepatitis C virus; PT: Prothrombin time; APTT: activated partial thromboplastin time; INR, International normalized ratio; MELD: model for end-stage liver disease; ALBI: albumin-bilirubin; PPIs: proton pump inhibitors; ¥: Renminbi.

## Data Availability

The data used to support the findings of this study are available from the corresponding author upon request.

## Abbreviations

AUGIB: Acute upper gastrointestinal bleeding
UGIB: Upper gastrointestinal bleeding
INR: International normalized ratio
PPIs: Proton pump inhibitors
MELD: Model for end-stage liver disease
ALBI: Albumin-bilirubin.

## Supplementary Materials

Supplementary Materials Supplementary Table 1. Treatment of cirrhotic patients with AUGIB at different departments from our hospital in test cohort. Supplementary Table 2. Subgroup analyses of patients with liver cirrhosis and AUGIB based on the origin of bleeding in test cohort. Supplementary Table 3. AUGIB patients with endoscopically confirmed varices after propensity matching analysis in test cohort.
