# Supplementary material for: Effect of Admission Time on the Outcomes of Liver Cirrhosis with Acute Upper Gastrointestinal Bleeding: Regular Hours versus Off-Hours Admission
Source: Can J Gastroenterol Hepatol. 2018 Nov 29;2018:3541365. doi: 10.1155/2018/3541365 (PMC6304553; doi:10.1155/2018/3541365)
Supplement: Supplementary Materials — Supplementary Table 1. Treatment of cirrhotic patients with AUGIB at different departments from our hospital in test cohort. Supplementary Table 2. Subgroup analyses of patients with liver cirrhosis and AUGIB based on the origin of bleeding in test cohort. Supplementary Table 3. AUGIB patients with endoscopically confirmed varices after propensity matching analysis in test cohort. [file 3541365.f1.docx]

| **Supplementary Table 1. Treatment of cirrhotic patients with AUGIB at different departments from our hospital in test cohort** | | | | |
| --- | --- | --- | --- | --- |
| **Departments** | **Overall** | **Regular hours admission** | **Off-hours admission** | **P value** |
| ***Department of Gastroenterology*** | 601 | 355 | 246 |  |
| Endoscopic Treatment | 350 (58.2%) | 221 (62.3%) | 129 (52.4%) | 0.016 |
| Somatostatin and/or Octreotide | 541 (90.0%) | 314 (88.5%) | 227 (92.3%) | 0.124 |
| Blood Transfusion | 402 (66.9%) | 222 (62.5%) | 180 (73.2%) | 0.006 |
| PPIs | 592 (98.5%) | 350 (98.6%) | 242 (98.4%) | 0.829 |
| Sengstaken Blakemore | 20 (3.3%) | 12 (3.4%) | 8 (3.3%) | 0.931 |
| ***Department of TCM*** | 118 | 67 | 51 |  |
| Endoscopic Treatment | 81 (68.6%) | 45 (67.2%) | 36 (70.6%) | 0.691 |
| Somatostatin and/or Octreotide | 114 (96.6%) | 64 (95.5%) | 50 (98.0%) | 0.454 |
| Blood Transfusion | 73 (61.9%) | 37 (55.2%) | 36 (70.6%) | 0.089 |
| PPIs | 117 (99.2%) | 66 (98.5%) | 51 (100%) | 0.381 |
| ***Department of Endoscopy*** | 59 | 39 | 20 |  |
| Endoscopic Treatment | 50 (84.7%) | 30 (76.9%) | 20 (100%) | 0.02 |
| Somatostatin and/or Octreotide | 56 (94.9%) | 36 (92.3%) | 20 (100%) | 0.203 |
| Blood Transfusion | 32 (54.2%) | 18 (46.2%) | 14 (70%) | 0.082 |
| PPIs | 57 (96.6%) | 37 (94.9%) | 20 (100%) | 0.303 |
| ***Department of Emergency*** | 24 | 13 | 11 |  |
| Endoscopic Treatment | 20 (83.3%) | 11 (84.6%) | 9 (81.8%) | 0.855 |
| Somatostatin and/or Octreotide | 22 (91.7%) | 12 (92.3%) | 10 (90.9%) | 0.902 |
| Blood Transfusion | 19 (79.2%) | 9 (69.2%) | 10 (90.9%) | 0.193 |
| PPIs | 23 (95.8%) | 12 (92.3%) | 11 (100%) | 0.347 |
| ***Department of Hepatobiliary Surgery*** | 17 | 8 | 9 |  |
| Endoscopic Treatment | 5 (29.4%) | 2 (25%) | 3 (33.3%) | 0.707 |
| Somatostatin and/or Octreotide | 14 (82.4%) | 7 (87.5%) | 7 (77.8%) | 0.6 |
| Blood Transfusion | 14 (82.4%) | 7 (87.5%) | 7 (77.8%) | 0.6 |
| PPIs | 17 (100%) | 8 (100%) | 9 (100%) | NA |
| Surgery | 8 (47.1%) | 2 (25%) | 6 (66.7%) | 0.086 |
| ***Other Departments*** | 7 | 4 | 3 |  |
| Endoscopic Treatment | 2 (28.6%) | 1 (25%) | 1 (33.3%) | 0.809 |
| Somatostatin and/or Octreotide | 3 (42.9%) | 2 (50%) | 1 (33.3%) | 0.659 |
| Blood Transfusion | 4 (57.1%) | 2 (50%) | 2 (66.7%) | 0.659 |
| PPIs | 7 (100%) | 4 (100%) | 3 (100%) | NA |
| Notes: AUGIB, acute upper gastrointestinal bleeding; PPIs, Proton pump inhibitors; TCM, Traditional Chinese medicine. | | | | |

| **Supplementary Table 2. Subgroup analyses of patients with liver cirrhosis and AUGIB based on the origin of bleeding in test cohort** | | | | | | | | | | |
| --- | --- | --- | --- | --- | --- | --- | --- | --- | --- | --- |
| **Variables** | **AUGIB with endoscopically confirmed varices (N=591)** | | | | **P value** | **AUGIB without varices at endoscopy (N=20)** | | | | **P value** |
|  | **No. Pts** | **Regular hours** | **No. Pts** | **Off-hours** |  | **No. Pts** | **Regular hours** | **No. Pts** | **Off-hours** |  |
| Age (Years) | 358 | 53.74 (20.88-81.62) | 233 | 55.81 (6.28-82.60) | 0.015 | 14 | 61.54 (50.48-83.67) | 6 | 63.12 (47.68-79.16) | 0.869 |
| Sex (Male) | 358 | 240 (67.0%) | 233 | 146 (62.7%) | 0.274 | 14 | 10 (71.4%) | 6 | 5 (83.3%) | 0.573 |
| Etiology of Liver Diseases | 358 |  | 233 |  | 0.449 | 14 |  | 6 |  | 0.158 |
| Viral Hepatitis |  | 124 (34.6%) |  | 73 (31.3%) |  |  | 6 (42.9%) |  | 5 (83.3%) |  |
| Alcohol Abuse |  | 85 (23.7%) |  | 61 (26.2%) |  |  | 2 (14.3%) |  |  |  |
| Viral Hepatitis + Alcohol Abuse |  | 41 (11.5%) |  | 23 (9.8%) |  |  |  |  |  |  |
| Drug Related |  | 20 (5.6%) |  | 10 (4.3%) |  |  | 1 (7.1%) |  |  |  |
| Autoimmune Liver Diseases |  | 17 (4.8%) |  | 19 (8.2%) |  |  | 2 (14.3%) |  |  |  |
| Unclear Etiology |  | 71 (19.8%) |  | 47 (20.2%) |  |  | 3 (21.4%) |  | 1 (16.7%) |  |
| Laboratory Tests |  |  |  |  |  |  |  |  |  |  |
| Red Blood Cell (10^12^/L) | 357 | 2.66 (0.98-5.49) | 233 | 2.48 (1.05-4.68) | 0.026 | 14 | 2.34 (1.25-3.64) | 6 | 2.86 (1.39-3.87) | 0.934 |
| Hemoglobin (g/L) | 357 | 74.00 (23.00-164.00) | 233 | 71.00 (23.00-142.00) | 0.249 | 14 | 68.50 (29.00-125.00) | 6 | 69.00 (40.00-100.00) | 0.409 |
| White Blood Cell (10^9^/L) | 357 | 3.90 (0.80-26.30) | 233 | 4.90 (1.00-46.10) | <0.001 | 14 | 7.45 (2.00-11.70) | 6 | 5.45 (1.60-12.40) | 0.433 |
| Platelet Count (10^9^/L) | 357 | 72.00 (9.00-548.00) | 233 | 71.00 (17.00-435.00) | 0.945 | 14 | 129.00 (49.00-163.00) | 6 | 121.50 (49.00-163.00) | 0.592 |
| Total Bilirubin (umol/L) | 355 | 19.80 (4.80-370.90) | 233 | 20.40 (3.30-187.40) | 0.404 | 14 | 17.65 (6.10-266.20) | 6 | 10.75 (4.80-22.00) | 0.409 |
| Direct Bilirubin (umol/L) | 355 | 7.70 (1.30-237.10) | 233 | 8.30 (0.50-151.60) | 0.763 | 14 | 7.35 (1.30-207.40) | 6 | 4.60 (2.80-8.50) | 0.409 |
| Indirect Bilirubin (umol/L) | 355 | 11.10 (2.40-133.80) | 233 | 11.70 (2.40-102.00) | 0.558 | 14 | 8.40 (3.60-58.80) | 6 | 6.80 (2.00-13.50) | 0.248 |
| Albumin (g/L) | 351 | 31.60 (10.00-49.30) | 226 | 29.20 (10.00-45.60) | <0.001 | 14 | 30.85 (23.70-48.40) | 6 | 33.05 (29.00-37.20) | 0.592 |
| Alanine Aminotransferase (U/L) | 354 | 22.00 (5.00-730.00) | 232 | 24.00 (5.00-1064.00) | 0.217 | 14 | 24.50 (12.00-95.00) | 6 | 34.50 (21.00-79.00) | 0.186 |
| Aspartate Aminotransferase (U/L) | 354 | 29.00 (7.00-1399.00) | 232 | 31.50 (9.00-1487.00) | 0.054 | 14 | 41.50 (12.00-773.00) | 6 | 38.50 (33.00-56.00) | 0.934 |
| Alkaline Phosphatase (U/L) | 354 | 71.00 (17.44-889.00) | 232 | 68.55 (1.30-688.00) | 0.465 | 14 | 78.45 (33.50-337.00) | 6 | 53.00 (49.00-93.00) | 0.058 |
| Gamma-glutamyl Transpeptidase (U/L) | 354 | 32.50 (5.00-1168.00) | 232 | 34.00 (8.00-755.00) | 0.921 | 14 | 98.50 (9.00-1126.00) | 6 | 28.00 (10.00-46.00) | 0.127 |
| Blood Urea Nitrogen (mmol/L) | 344 | 6.48 (1.58-42.83) | 225 | 8.06 (1.96-28.25) | <0.001 | 14 | 11.35 (3.16-35.71) | 6 | 6.95 (4.18-29.39 | 0.934 |
| Serum Creatinine (umol/L) | 344 | 58.40 (20.00-715.00) | 224 | 61.00 (24.00-327.00) | 0.058 | 14 | 62.50 (33.00-449.00) | 6 | 60.00 (37.00-92.20) | 0.68 |
| Potassium (mmol/L) | 350 | 4.00 (2.13-5.50) | 231 | 4.10 (2.86-6.85) | 0.001 | 14 | 3.87 (3.22-5.27) | 6 | 3.85 (3.49-4.14) | 0.837 |
| Sodium (mmol/L) | 350 | 139.05 (83.00-160.80) | 231 | 138.50 (121.30-150.00) | 0.259 | 14 | 141.10 (134.00-151.40) | 6 | 139.50 (133.90-142.80) | 0.16 |
| PT (seconds) | 345 | 15.70 (10.80-62.80) | 226 | 16.30 (11.00-46.30) | 0.027 | 13 | 14.50 (12.50-47.00) | 5 | 14.50 (13.30-49.50) | 0.622 |
| APTT (seconds) | 343 | 40.10 (25.70-180.00) | 225 | 39.40 (27.30-97.20) | 0.438 | 13 | 36.80 (30.00-51.80) | 5 | 33.50 (27.30-51.00) | 0.588 |
| INR | 343 | 1.25 (0.77-7.96) | 225 | 1.32 (0.79-4.77) | 0.04 | 13 | 1.14 (0.93-5.21) | 5 | 1.12 (1.02-5.94) | 0.587 |
| Child-Pugh Score | 338 | 7 (5-15) | 219 | 7 (5-15) | <0.001 | 13 | 7 (5-11) | 5 | 6 (5-10) | 0.244 |
| Child-Pugh A/B/C | 338 | 137 (40.5%)/162 (47.9%)/39 (11.5%) | 219 | 53 (24.2%)/128 (58.4%)/38 (17.4%) | <0.001 | 13 | 6 (46.2%)/4  (30.8%)/3 (23.1%) | 5 | 4 (80.0%)/0  (0%)/1 (20.0%) | 0.317 |
| MELD Score | 336 | 5.30 (-6.50-28.87) | 218 | 6.49 (-7.44-37.65) | 0.023 | 13 | 4.27 (-5.33-32.06) | 5 | 1.53 (0.11-18.70) | 0.805 |
| Recalibrated MELD Score | 336 | -4.21 (-6.66-0.66) | 218 | -3.97 (-6.85-2.48) | 0.023 | 13 | -4.43 (-6.42-1.32) | 5 | -4.99 (-5.29- -1.44) | 0.805 |
| ALBI Score | 346 | -1.83 (-3.23- -0.08) | 223 | -1.58 (-3.06-0.22) | <0.001 | 14 | -1.90 (-3.26- -0.91) | 6 | -2.08 (-2.50- -1.88) | 0.284 |
| Treatment |  |  |  |  |  |  |  |  |  |  |
| Endoscopic Treatment | 358 | 306 (85.5%) | 233 | 193 (82.8%) | 0.387 | 14 | 1 (7.1%) | 6 | 1 (16.7%) | 0.515 |
| Sengstaken Blakemore | 358 | 9 (2.5%) | 233 | 5 (2.1%) | 0.774 | 14 | 0 (0%) | 6 | 0 (0%) | NA |
| Somatostatin and/or Octreotide | 358 | 341 (95.3%) | 233 | 223 (95.7%) | 0.795 | 14 | 9 (64.3%) | 6 | 5 (83.3%) | 0.394 |
| Blood Transfusion | 358 | 213 (59.5%) | 233 | 176 (75.5%) | <0.001 | 14 | 7 (50.0%) | 6 | 3 (50.0%) | 1 |
| PPIs | 358 | 355 (99.2%) | 233 | 232 (99.6%) | 0.554 | 14 | 14 (100%) | 6 | 6 (100%) | NA |
| Surgery | 358 | 1 (0.3%) | 233 | 5 (2.1%) | 0.027 | 14 | 0 (0%) | 6 | 0 (0%) | NA |
| 5-day Re-bleeding After Treatment | 357 | 38 (10.6%) | 230 | 38 (16.5%) | 0.038 | 14 | 0 (0%) | 6 | 1 (16.7%) | 0.117 |
| Death During Hospitalization | 358 | 8 (2.2%) | 233 | 11 (4.7%) | 0.094 | 14 | 0 (0%) | 6 | 0 (0%) | NA |
| Length of Stay (days) | 358 | 11.95 (0.16-78.00) | 233 | 11.65 (0.25-57.93) | 0.856 | 14 | 8.04 (1.03-16.99) | 6 | 8.15 (3.68-53.64) | 0.869 |
| Total Payments (¥) | 358 | 23,864.24 (2,116.91-126,413.58) | 233 | 29,361.51 (3,877.92-226,872.93) | <0.001 | 14 | 9,965.34 (3,632.41-23,878.04) | 6 | 17,125.80 (6,681.50-117,223.08) | 0.187 |
| Data are expressed as median (range) or frequency (percentage).  Note: AUGIB, acute upper gastrointestinal bleeding; PT, Prothrombin time; APTT, Activated partial thromboplastin time; INR, International normalized ratio; MELD, Model for end-stage liver disease; ALBI, Albumin-Bilirubin; PPIs, Proton pump inhibitors; ¥, Renminbi. | | | | | | | | | | |

| **Supplementary Table 3. AUGIB patients with endoscopically confirmed varices after propensity matching analysis in test cohort** | | | |
| --- | --- | --- | --- |
| **Variables** | **Regular hours (N=140)** | **Off-hours (N=140)** | **P value** |
| Age (Years) | 53.05 (29.08-80.05) | 55.07 (6.28-77.71) | 0.256 |
| Sex (Male) | 90 (64.3%) | 94 (67.1%) | 0.615 |
| Etiology of Liver Diseases |  |  | 0.09 |
| Viral Hepatitis | 56 (40.0%) | 47 (33.6%) |  |
| Alcohol Abuse | 26 (18.6%) | 38 (27.1%) |  |
| Viral Hepatitis + Alcohol Abuse | 17 (12.1%) | 10 (7.1%) |  |
| Drug Related | 12 (8.6%) | 6 (4.3%) |  |
| Autoimmune Liver Diseases | 9 (6.4%) | 12 (8.6%) |  |
| Unclear Etiology | 20 (14.3%) | 27 (19.3%) |  |
| Laboratory Tests |  |  |  |
| Red Blood Cell (10^12^/L) | 2.59 (0.98-4.38) | 2.49 (1.05-4.68) | 0.506 |
| Hemoglobin (g/L) | 72.50 (23.00-137.00) | 71.00 (23.00-133.00) | 0.78 |
| White Blood Cell (10^9^/L) | 3.60 (1.10-26.30) | 4.55 (1.00-46.10) | 0.007 |
| Platelet Count (10^9^/L) | 68.50 (15.00-548.00) | 73.50 (17.00-435.00) | 0.353 |
| Total Bilirubin (umol/L) | 19.95 (5.30-196.10) | 19.85 (5.20-187.40) | 0.682 |
| Direct Bilirubin (umol/L) | 8.20 (1.50-169.70) | 7.70 (1.60-151.60) | 0.158 |
| Indirect Bilirubin (umol/L) | 11.40 (3.20-71.70) | 11.75 (2.70-69.90) | 0.82 |
| Albumin (g/L) | 30.90 (10.50-45.10) | 30.25 (10.00-45.60) | 0.349 |
| Alanine Aminotransferase (U/L) | 20.50 (8.00-730.00) | 23.00 (5.00-1064.00) | 0.566 |
| Aspartate Aminotransferase (U/L) | 29.00 (8.00-1399.00) | 30.00 (9.00-1095.00) | 0.463 |
| Alkaline Phosphatase (U/L) | 73.25 (35.00-493.00) | 67.00 (20.00-688.00) | 0.192 |
| Gamma-glutamyl Transpeptidase (U/L) | 32.00 (8.00-475.00) | 32.00 (9.00-737.00) | 0.645 |
| Blood Urea Nitrogen (mmol/L) | 6.45 (1.58-28.90) | 7.83 (1.96-28.25) | 0.01 |
| Serum Creatinine (umol/L) | 57.00 (20.00-250.00) | 62.00 (24.00-304.00) | 0.178 |
| Potassium (mmol/L) | 4.00 (2.78-5.12) | 4.15 (2.86-6.85) | 0.001 |
| Sodium (mmol/L) | 139.25 (83.00-147.30) | 138.55 (128.00-150.00) | 0.385 |
| PT (seconds) | 15.80 (10.80-62.80) | 16.25 (11.00-36.40) | 0.424 |
| APTT (seconds) | 40.75 (27.80-180.00) | 39.10 (27.30-96.60) | 0.218 |
| INR | 1.26 (0.77-7.96) | 1.31 (0.79-3.52) | 0.45 |
| Child-Pugh Score | 7 (5-13) | 7 (5-12) | 0.952 |
| Child-Pugh A/B/C | 47 (33.6%)/70 (50.0%)/23 (16.4%) | 45 (32.1%)/72 (51.4%)/23 (16.4%) | 0.965 |
| MELD Score | 5.39 (-4.57-26.48) | 6.24 (-7.44-26.11) | 0.372 |
| Recalibrated MELD Score | -4.21 (-6.66-0.66) | -3.97 (-6.85-2.48) | 0.372 |
| ALBI Score | -1.77 (-3.03- -0.08) | -1.69 (-3.06-0.22) | 0.44 |
| Treatment |  |  |  |
| Endoscopic Treatment | 118 (84.3%) | 119 (85.0%) | 0.868 |
| Sengstaken Blakemore | 4 (2.9%) | 3 (2.1%) | 0.702 |
| Somatostatin and/or Octreotide | 133 (95.0%) | 133 (95.0%) | 1 |
| Blood Transfusion | 82 (58.6%) | 108 (77.1%) | 0.001 |
| PPIs | 139 (99.3%) | 139 (99.3%) | 1 |
| Surgery | 0 (0%) | 4 (2.9%) | 0.044 |
| 5-day Re-bleeding After Treatment | 17 (12.1%) | 23 (16.4%) | 0.306 |
| Death During Hospitalization | 2 (1.4%) | 4 (2.9%) | 0.409 |
| Length of Stay (days) | 12.91 (2.78-54.00) | 11.75 (0.25-57.93) | 0.421 |
| Total Payments (¥) | 25,914.13 (2,776.55-115,201.67) | 29,068.03 (4,203.51-143,048.30) | 0.058 |
| Data are expressed as median (range) or frequency (percentage).  Note: AUGIB, acute upper gastrointestinal bleeding; PT, Prothrombin time; APTT, Activated partial thromboplastin time; INR, International normalized ratio; MELD, Model for end-stage liver disease; ALBI, Albumin-Bilirubin; PPIs, Proton pump inhibitors; ¥, Renminbi. | | | |
